# Four-Year Longitudinal Study of Motor and Non-motor Symptoms in *LRRK2*-Related Parkinson's Disease

**DOI:** 10.3389/fneur.2019.01379

**Published:** 2020-01-17

**Authors:** Xiao Deng, Bin Xiao, Hui-Hua Li, Ebonne Ng, Yew-Long Lo, Eng-King Tan, Kumar M. Prakash

**Affiliations:** ^1^Department of Neurology, National Neuroscience Institute, Singapore General Hospital, Singapore, Singapore; ^2^Health Services Research Unit, Singapore General Hospital, Singapore, Singapore; ^3^Signature Research Program in Neuroscience and Behavioral Disorders, Duke-NUS Graduate Medical School, Singapore, Singapore

**Keywords:** Parkinson's disease, progression, *LRRK2*, motor, non-motor

## Abstract

**Objectives:** In a prospective 4-year study, we evaluated the progression of motor and non-motor symptoms in Parkinson's disease (PD) patients with Asian-specific *LRRK2* risk variants and non-carriers.

**Methods:** A total of 202 patients with PD, including 133 risk variant carriers and 69 non-carriers, were followed up and evaluated using the Modified Hoehn and Yahr staging scale, Unified Parkinson's Disease Rating Scale part III, Non-motor Symptom Scale, Parkinson's disease Questionnaire-39 item version. Means of generalized estimating equation model was performed to compare the differences from baseline between *LRRK2* risk variant carriers and non-carriers.

**Results:** Our longitudinal analysis revealed that risk variant carriers exhibited greater progression than non-carriers after 4 years based on the modified Hoehn and Yahr staging scale (risk variants carriers, 0.65; non-carriers, 0.06; *P* = 0.041). Meanwhile, Unified Parkinson's Disease Rating Scale gait and posture score in risk variant carriers also showed greater increase than that in non-carriers, although the difference was not statistically significant. Non-carriers experienced a transient improvement in non-motor symptoms at the early stage of PD, as scores at visit two significantly reduced compared to baseline in Non-motor Symptom Scale domain 3 (mood/apathy), Parkinson's disease Questionnaire-39 item version domain 3 (emotional well-being), and frequency of NMS in non-carriers but not in risk variants carriers.

**Conclusions:** PD gene risk variant carriers were more likely to progress faster in their motor severity than non-carriers. There were transient differences in certain non-motor symptoms and quality of life in carriers. However, more studies are warranted to assess the association of PD risk variants and progression of non-motor symptoms.

## Introduction

PD is a common neurodegenerative disease with clinical heterogeneity and diverse causative factors. At least nine genes have been implicated in PD, with overlapping pathological features (1). The common *LRRK2* G2019S mutation is located in the kinase domain, possibly associated with an increase in kinase activity (2). Clinically, patients harboring G2019S share considerable similarity with sporadic PD patients (3). The polymorphic variants G2385R, R1628P, and S1647T are associated with increased risk for PD in the Asian population (4).

A recent study showed that PD patients with the Asian *LRRK2* variants experienced greater progression in motor symptoms (5). However, other aspects of the disease, including non-motor symptoms which greatly account for the decline of quality of life in PD, were not evaluated during the follow-up. To address this gap in knowledge, we conducted a longitudinal study to evaluate the progression of both motor and non-motor features in Asian *LRRK2* PD carriers compared to non-carriers.

## Methods

### Study Population

PD patients were assessed prospectively from movement disorder outpatient clinics in Singapore General Hospital over a 4-year period. The clinical diagnosis was based on the UK PD Brain Bank criteria (6). PD patients who received deep brain stimulation surgery or whose PD diagnosis was amended during the follow-up period were excluded. Our study was approved by SingHealth Centralized Institutional Review Board, and all methods were performed in accordance with the relevant guidelines and regulations. Written informed consent was obtained from all subjects.

### Genetic Analyses and Grouping Patients

Three *LRRK2* variants (R1628P, S1647T, and G2385R) were genotyped by TaqMan real-time PCR. Based on the genotyping results, we grouped the patients into *LRRK2* risk variant carriers (R1628P, S1647T, and/or G2385R missense variants) and non-carriers (wild type for all the *LRRK2* variants).

### Data Collection and Assessments

Demographic data were collected from all subjects. The levodopa equivalent daily dose was calculated by standardized formula (7). Modified Hoehn and Yahr (H&Y) staging scale, Unified Parkinson's Disease Rating Scale (UPDRS) part III, Non-motor Symptom Scale (NMSS), Parkinson's disease Questionnaire-39 item version (PDQ-39), and Elderly Cognitive Assessment Questionnaire were applied to assess disease severity, motor symptoms, non-motor burden, quality of life, and cognitive impairment, respectively. UPDRS scores were obtained during the “on” medication period. There are nine domains in NMSS, including cardiovascular domain, sleep/fatigue, mood/apathy, perceptual problems/hallucinations, attention/memory, gastrointestinal, urinary, sexual function, and miscellaneous (8). The overall non-motor burden (NMSS total score) is calculated by the sum of all the domain scores. PDQ-39 comprises 39 questions categorized in 8 domains (mobility, activities of daily living, emotional well-being, stigma, social support, cognitive impairment, communication, and bodily discomfort). Summary index (SI) is calculated for the total PDQ-39 scale (PDQ-39 SI) and the eight domains. A higher NMSS score and PDQ-39 SI indicate greater NMS burden and worse quality of life. A lower Elderly Cognitive Assessment Questionnaire score suggests greater cognitive impairment. The assessments were conducted by one clinical research staff with formalized training on the same day at baseline and follow-up visits. The follow-up visits were carried out yearly, and the final visit was up to 4 years. Both the rater and patients were blinded to genotype status.

### Statistical Analysis

Frequency together with proportion was reported for categorical data, while median together with interquartile range was reported for continuous variables at baseline. Fisher's exact test was performed to compare categorical data at baseline among different groups of patients, while the Mann–Whitney test was carried out to compare the distribution of these continuous variables between different groups of patients. The differences from baseline in patients with risk variants were compared to the control group by means of generalized estimating equation model. Each score was standardized; an interaction term between visit and group was examined for its significance by means of GEE model. Data analysis was performed in Stata V13.1 (Stata Corp, College Station, TX, USA) and R 3.1.3 (www.r-project.org) with significance level of 5%.

## Results

A total of 202 PD patients, including 133 patients with risk variants and 69 controls without risk variants, were assessed prospectively over a 4-year period. One hundred thirty-three patients carried at least one of the three risk variants. The G2385R frequency was 6.8% (17 patients), the R1628P frequency was 8.4% (21 patients), while the S1647T frequency was 53.0% (132 patients). At baseline, there were no significant differences in the demographic characteristics, UPDRS score, and NMSS score between risk variant carriers and non-carriers (Table 1).

**Table 1:** Comparison of demographic and baseline clinical data between two groups.

	**Non-carriers** **(*n* = 69)**	**Risk variants** **(*n* = 133)**	***P*-value**
Age of onset	57.5 (50, 64.5)	60 (53, 66)	0.3549
Gender			0.2867
Male	39 (56.5%)	86 (64.7%)	
Female	30 (43.5%)	47 (35.3%)	
Ethnicity			0.5691
Chinese	60 (87%)	120 (90.2%)	
Non-Chinese	9 (13%)	13 (9.8%)	
PD family history			0.1247
No	66 (95.7%)	117 (88.0%)	
Yes	3 (4.3%)	16 (12.0%)	
Young onset			0.5712
No	54 (79.4%)	109 (82.6%)	
Yes	14 (20.6%)	23 (17.4%)	
Disease duration	5 (3, 8)	6 (3, 9)	0.2767
Education years	8.6 ± 5.0	8.3 ± 4.8	0.1730
LEDD (mg/day)	300 (150, 540)	399 (200, 550)	0.4021
UPDRS total score	28 (10, 36)	29 (19, 38)	0.1780
UPDRS tremor score	1 (0, 3)	2 (0, 4.5)	0.4662
UPDRS postural and gait score	4 (2, 6)	4 (3, 6)	0.2616
H&Y [median (IQR)]	2.5 (1.5, 2.5)	2.5 (2, 2.5)	0.1747
NMSS total score	22 (12, 44)	26 (14, 52)	0.2048
ADL scale	90 (80, 90)	80 (70, 90)	0.0874
HAMD	5 (2, 7)	4 (2, 9)	0.8217

*LEDD, levodopa equivalent daily dose; UPDRS, Unified Parkinson's Disease Rating Scale; NMS, non-motor symptom; H&Y, Hoehn and Yahr staging scale; IQR, interquartile range; ADL scale, Schwab and England activities of daily living scale; HAMD, Hamilton Depression Rating Scale; PD Family History, patients have positive family history of Parkinson's disease; Young onset, onset age of PD patients is <50 years old*.

**Figure 1:**
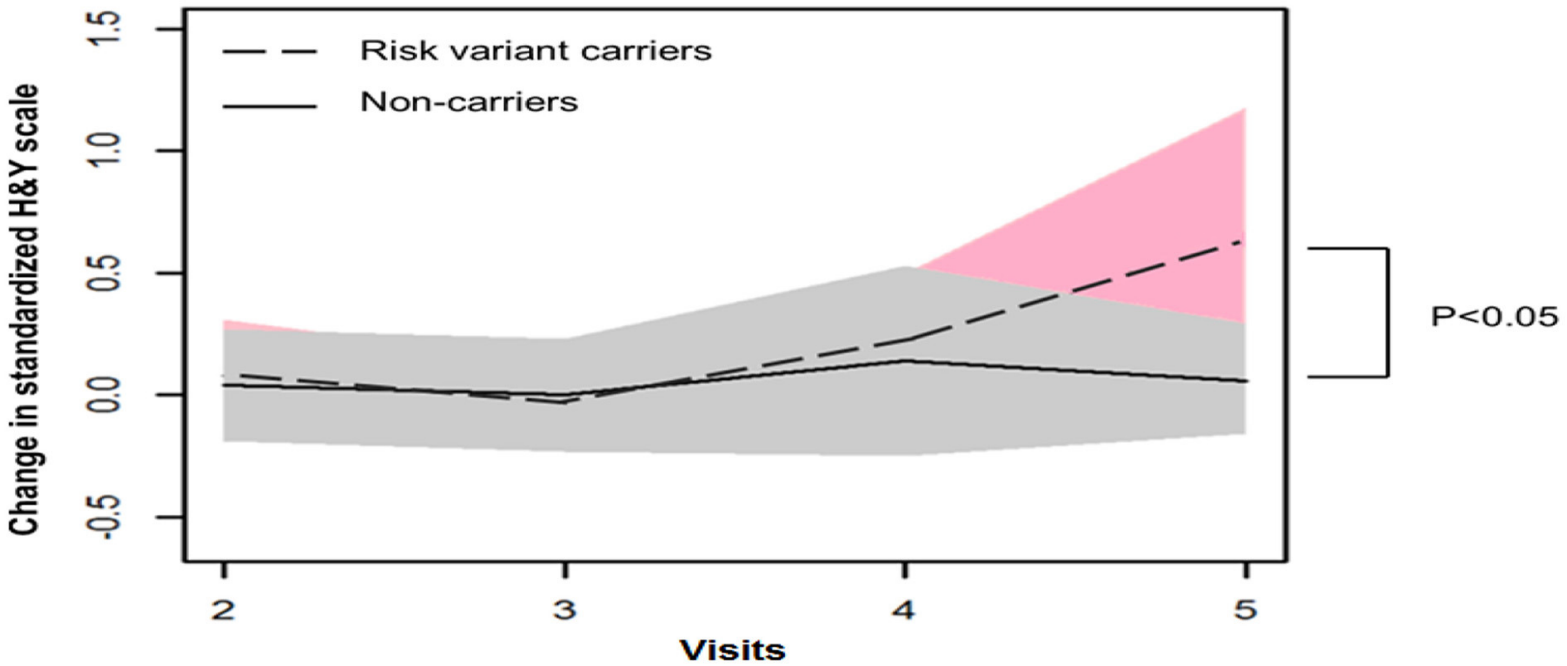
Progression of Hoehn and Yahr (H&Y) score in two groups.

**Figure 2:**
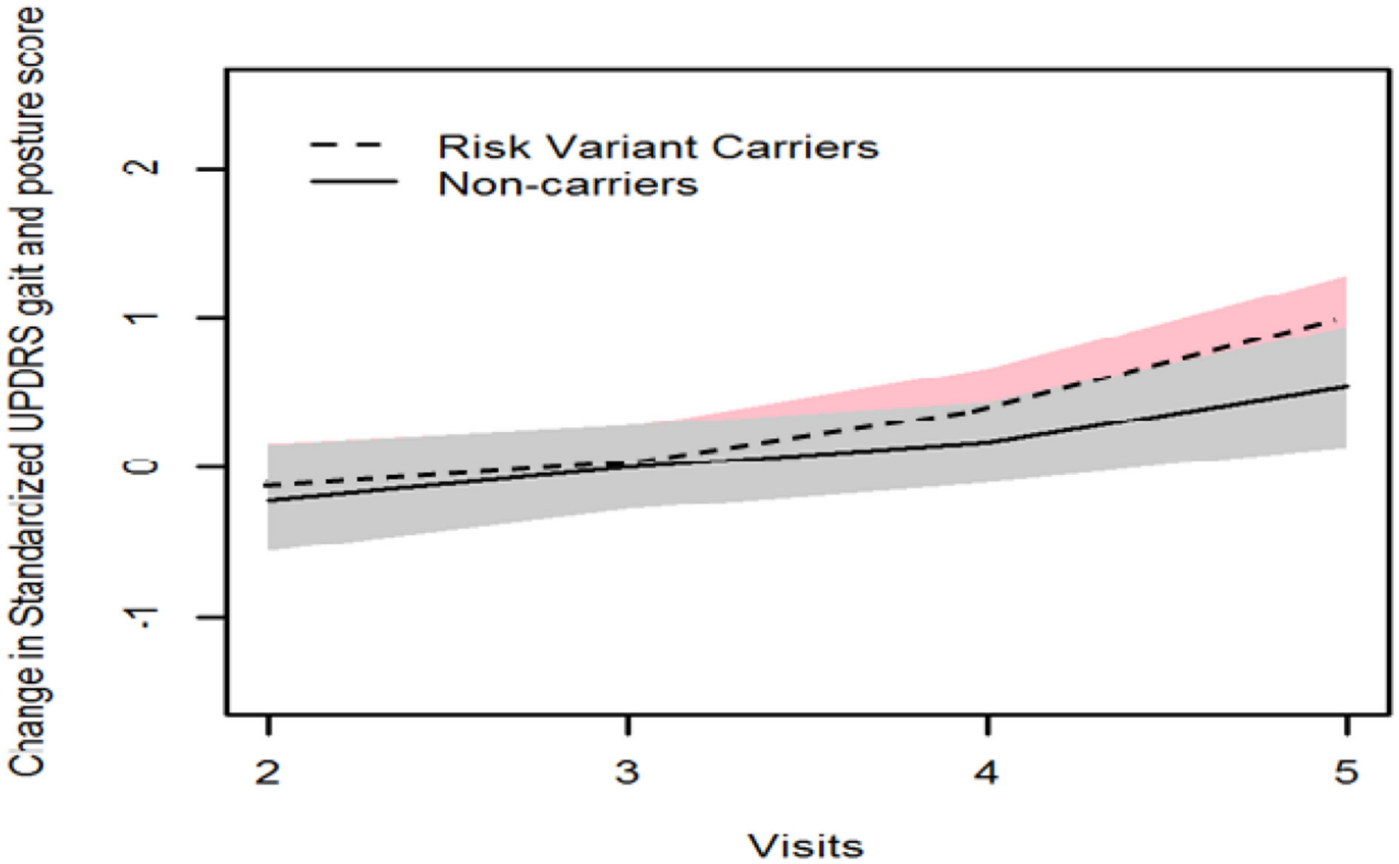
Progression of UPDRS posture and gait score in two groups.

GEE model analysis showed that mean H&Y scale increased more substantially after 4 years onwards in patients with risk variants (risk variants carriers, 0.65; non-carriers, 0.06, *P* = 0.041). Consistently, risk variant carriers showed faster progression of UPDRS postural and gait score than non-carriers from baseline to year 4 follow-up (visit 5), although the differences were not significant (risk variants carriers, 0.83; non-carriers, 0.54, *P* = 0.348) (Table 2, Figures 1, 2).

**Table 2:** Comparison of mean change from baseline in selected parameters between two groups by generalized estimating equation (GEE) model.

**Time period**	**Non-carriers**	**Risk variants**	***P*-value**
**Standardized UPDRS total score**			
Change at visit 2	−0.30 (−0.68, 0.07)	−0.02 (−0.25, 0.21)	0.202
Change at visit 3	0.07 (−0.19, 0.34)	0.09 (−0.06, 0.24)	0.910
Change at visit 4	0.40 (0.17, 0.62)	0.43 (0.21, 0.64)	0.797
Change at visit 5	0.69 (0.31, 1.07)	0.71 (0.34, 1.07)	0.945
**Standardized UPDRS posture and gait score**			
Change at visit 2	−0.21 (−0.56, 0.15)	−0.09 (−0.34, 0.16)	0.582
Change at visit 3	0 (−0.28, 0.28)	0.10 (−0.06, 0.27)	0.538
Change at visit 4	0.17 (−0.10, 0.44)	0.37 (0.09, 0.66)	0.278
Change at visit 5	0.54 (0.13, 0.94)	0.83 (0.38, 1.28)	0.348
**Standardized H&Y scale**			
Change at visit 2	0.04 (−0.19, 0.27)	0.08 (−0.14, 0.31)	0.800
Change at visit 3	0 (−0.23, 0.23)	−0.04 (−0.21, 0.13)	0.803
Change at visit 4	0.14 (−0.25, 0.53)	0.20 (−0.1, 0.50)	0.835
Change at visit 5	0.06 (−0.16, 0.29)	0.65 (0.13, 1.18)	**0.041**
**Standardized frequency of NMS**			
Change at visit 2	−0.65 (−0.95, −0.34)	−0.16 (−0.48, 0.16)	**0.035**
Change at visit 3	−0.14 (−0.45, 0.18)	0.08 (−0.12, 0.28)	0.269
Change at visit 4	0.57 (0.23, 0.91)	0.51 (0.28, 0.74)	0.772
Change at visit 5	0.95 (0.51, 1.39)	0.94 (0.50, 1.38)	0.952
**Standardized NMSS domain 3 score**			
Change at visit 2	−0.51 (−0.76, −0.26)	−0.15 (−0.39, 0.08)	**0.042**
Change at visit 3	−0.22 (−0.47, 0.03)	−0.2 (−0.38, −0.02)	0.879
Change at visit 4	0.39 (−0.22, 0.99)	0.33 (0.02, 0.64)	0.869
Change at visit 5	0.59 (0.14, 1.05)	1.09 (0.43, 1.75)	0.219
**Standardized PDQ-39 domain 3 SI**			
Change at visit 2	−0.25 (−0.51, 0.01)	0.21 (−0.11, 0.53)	**0.029**
Change at visit 3	0.04 (−0.23, 0.31)	0.07 (−0.15, 0.3)	0.853
Change at visit 4	0.65 (0.12, 1.18)	0.54 (0.22, 0.86)	0.729
Change at visit 5	1.06 (0.45, 1.68)	1.56 (0.9, 2.21)	0.286

*Vaues in bold indicate significant difference*.

*Frequency of NMS, total number of NMS items; NMSS domain 3, mood/apathy; PDQ-39, Parkinson's disease questionnaire-39 item version; PDQ-39 domain 3, emotional well-being*.

**Figure 3:**
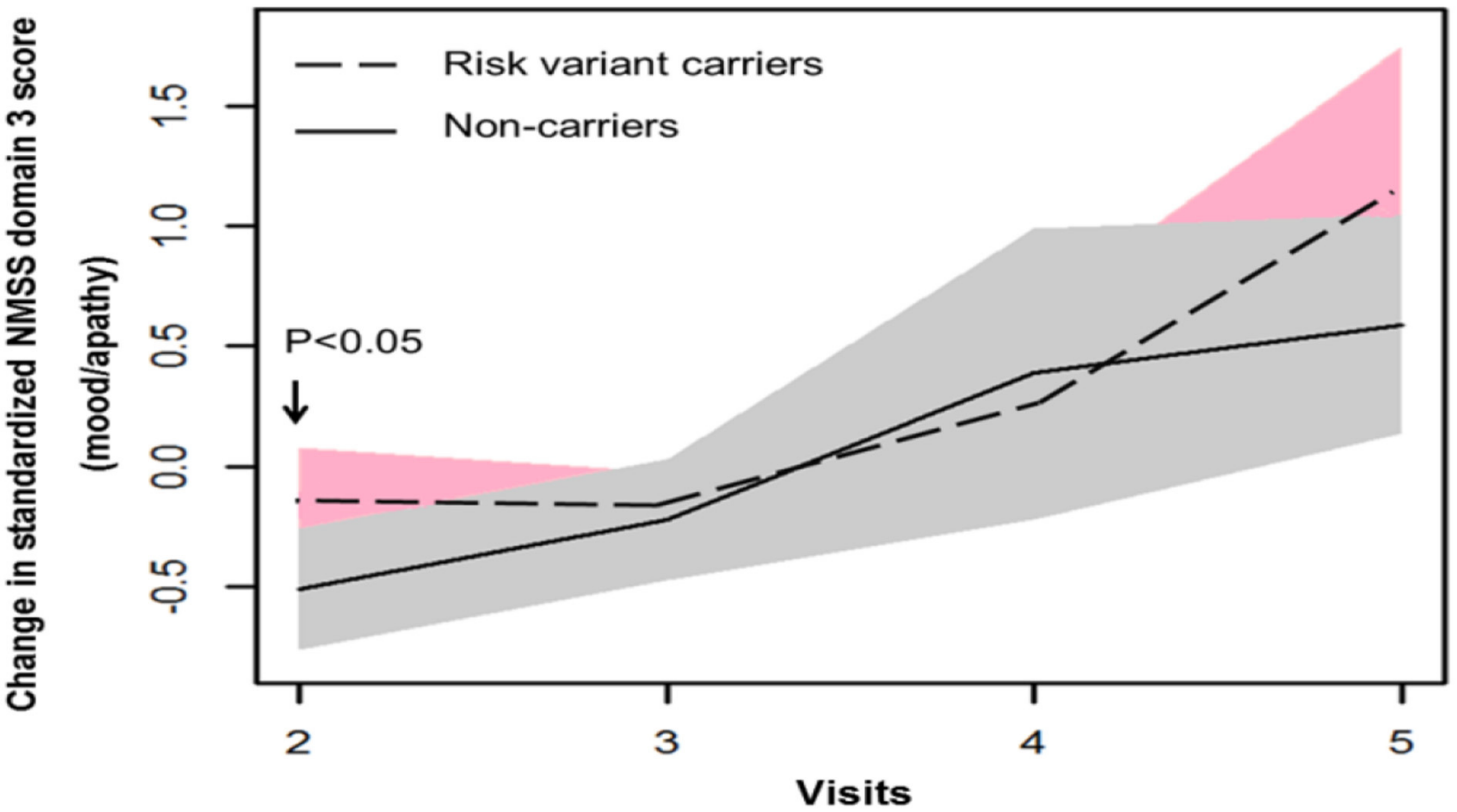
Progression of Non-motor Symptom Scale (NMSS) domain 3 score in two groups.

**Figure 4:**
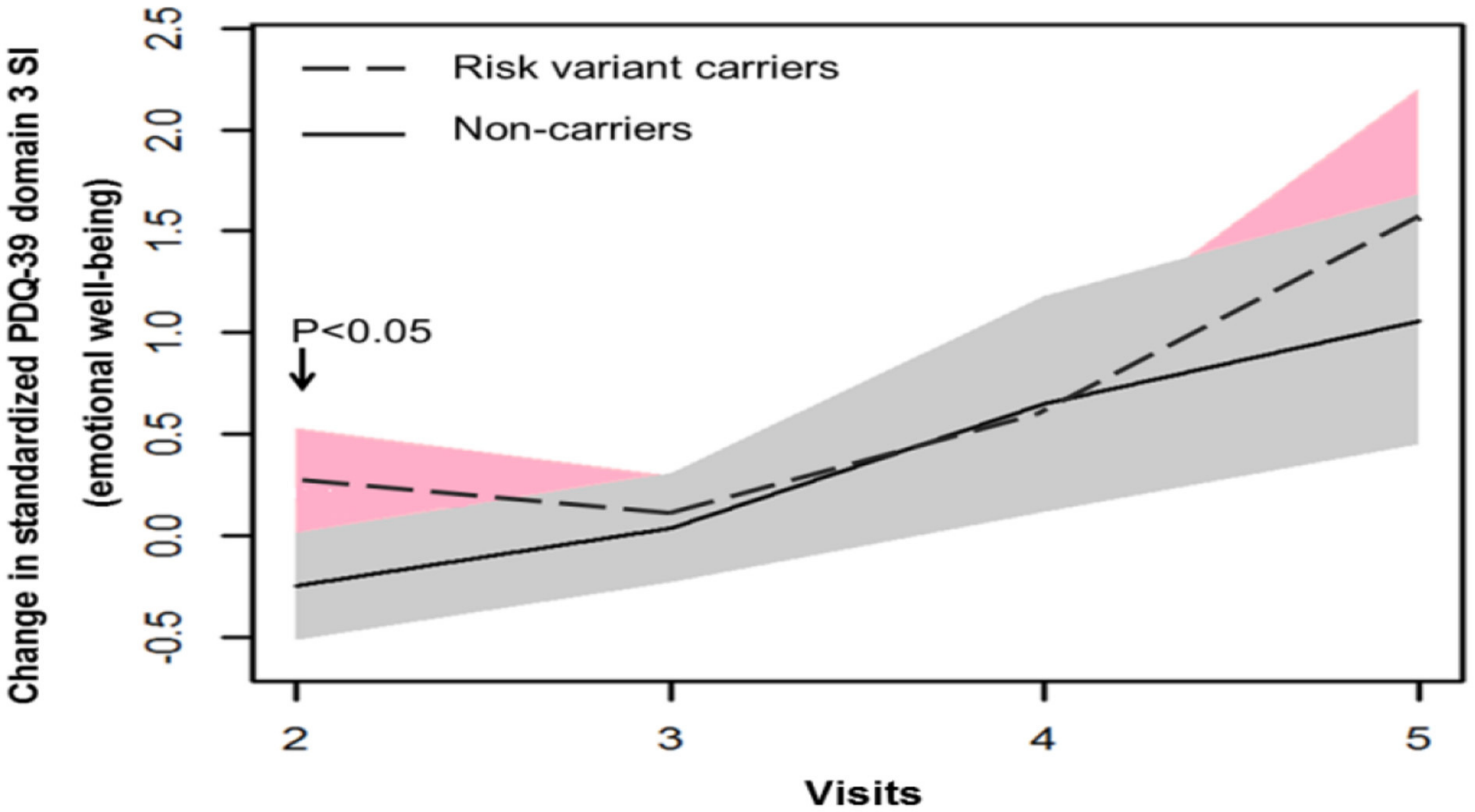
Progression of Parkinson's disease Questionnaire-39 item version (PDQ-39) domain 3 score in two groups.

In terms of non-motor symptoms, at year 1 follow-up (visit 2), scores of non-carriers in mean NMSS domain 3 score (mood/apathy), mean PDQ-39 domain 3 SI (emotional well-being), and total number of NMS items were simultaneously reduced. These score changes were significantly different between non-carriers and risk variant carriers (Table 2, Figures 3, 4). However, the progression of non-motor symptoms was not significantly different after year 1 between the two groups.

## Discussion

Our long-term follow-up revealed greater disease severity after 4 years onwards using Modified H&Y staging scale, which grades the overall motor function of the patients. In particular, postural instability contributes significantly to the scores in H&Y staging (9). Consistently, the trend is notable that patients with risk variants in our study present with higher UPDRS postural and gait score compared with non-carriers, although there was no statistical significance probably due to limited sample size. It is also possible that risk variant carriers would show more substantial motor progression if the follow-up duration were to be extended. In line with our hypothesis, Oosterveld et al. reported that PD patients in another independent cohort who harbored the same *LRRK2* risk variant(s) presented greater motor progression after 4 years onwards (5). It was reported that *LRRK2* G2385R variant carriers presented more severe form of PD compared with G2019S and idiopathic PD patients (10). Therefore, specific *LRRK2* variants should be analyzed separately in clinical studies. However, it is not statistically feasible in our study due to insufficient subjects in each subgroup. In addition, when studying the effect of *LRRK2* variant on PD progression, it is optimal to examine the status of other PD-related genes, which may be associated with *LRRK2* and affect the risk of *LRRK2*-related PD, such as *SNCA* (11) and *GBA* (3).

In terms of non-motor symptoms and quality of life, transient differences were found in NMS total frequency, NMSS domain 3 (mood and apathy), and PDQ-39 domain 3 (emotional well-being) between non-carriers and risk variant carriers at early stage of the disease. This indicates progression course of non-motor symptoms and quality of life in PD with risk variants may differ from non-carriers. These non-motor symptoms may have to be identified and addressed timely in PD patients carrying risk variants.

Our current prospective study provides comprehensive information on the progression of multifaceted clinical features in PD with Asian-related risk variants, including motor, non-motor symptoms, and quality of life. Our study found that progression of motor symptoms became faster in risk variant carriers after 4 years, whereas progression of non-motor symptoms differed at early stage of disease between non-carriers and risk variant carriers, suggesting that *LRRK2* carriers may need to be closely monitored and managed for clinical progression.

We acknowledge that sample size may be a potential limitation in our study, which may increase the risk of false positive error. Validation of our findings in larger cohort will be warranted.

## Data Availability Statement

The datasets during the current study available from the corresponding author on reasonable request.

## Ethics Statement

The studies involving human participants were reviewed and approved by Sing health Centralized Institutional Review Board. The patients/participants provided their written informed consent to participate in this study.

## Author Contributions

E-KT, KP, XD, and BX designed the study. XD and BX wrote the main manuscript text. XD prepared all the tables. XD and H-HL conducted the statistical analysis. E-KT and KP gave revision of the manuscript. E-KT, XD, BX, EN, Y-LL, and KP conducted the research project. All authors reviewed the manuscript.

### Conflict of Interest

The authors declare that the research was conducted in the absence of any commercial or financial relationships that could be construed as a potential conflict of interest.
